# Role of Stem Cells in Pathophysiology and Therapy of Spondyloarthropathies—New Therapeutic Possibilities?

**DOI:** 10.3390/ijms19010080

**Published:** 2017-12-28

**Authors:** Magdalena Krajewska-Włodarczyk, Agnieszka Owczarczyk-Saczonek, Waldemar Placek, Adam Osowski, Piotr Engelgardt, Joanna Wojtkiewicz

**Affiliations:** 1 Department of Rheumatology, Municipal Hospital in Olsztyn, 10-900 Olsztyn, Poland; 2 Department of Pathophysiology, Faculty of Medicine, University of Warmia and Mazury, 10-900 Olsztyn, Poland; adam.osowski@uwm.edu.pl; 3 Department of Dermatology, Sexually Transmitted Diseases and Clinical Immunology, Faculty of Medicine, University of Warmia and Mazury, 10-900 Olsztyn, Poland; w.placek@wp.pl; 4 Department of Forensic Medicine, Faculty of Medicine, University of Warmia and Mazury, 10-900 Olsztyn, Poland; ra-bit@wp.pl; 5 Laboratory for Regenerative Medicine, Faculty of Medicine, University of Warmia and Mazury, 10-900 Olsztyn, Poland; 6 Foundation for Nerve Cell Regeneration, University of Warmia and Mazury in Olsztyn, 10-900 Olsztyn, Poland

**Keywords:** spondyloarthropathies, inflammation, mesenchymal stem cells

## Abstract

Considerable progress has been made recently in understanding the complex pathogenesis and treatment of spondyloarthropathies (SpA). Currently, along with traditional disease modifying anti-rheumatic drugs (DMARDs), TNF-α, IL-12/23 and IL-17 are available for treatment of such diseases as ankylosing spondylitis (AS) and psoriatic arthritis (PsA). Although they adequately control inflammatory symptoms, they do not affect the abnormal bone formation processes associated with SpA. However, the traditional therapeutic approach does not cover the regenerative treatment of damaged tissues. In this regards, stem cells may offer a promising, safe and effective therapeutic option. The aim of this paper is to present the role of mesenchymal stromal cells (MSC) in pathogenesis of SpA and to highlight the opportunities for using stem cells in regenerative processes and in the treatment of inflammatory changes in articular structures.

## 1. Introduction

**Figure 1:**
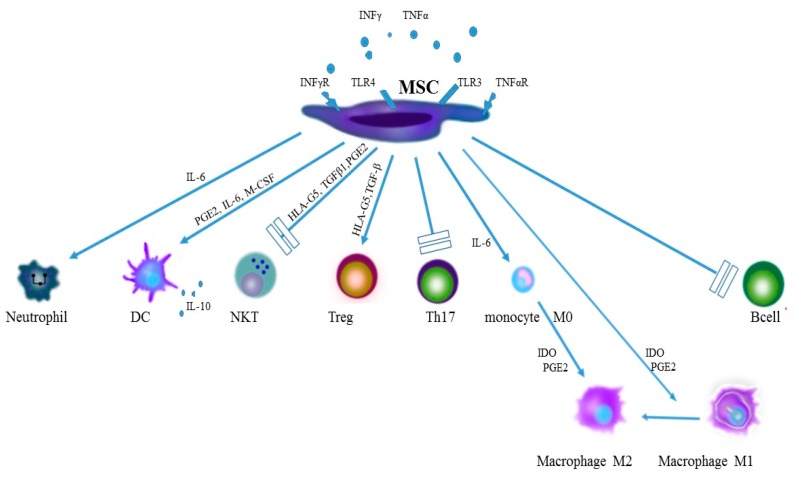
Immunomodulatory effect of MSC on elements of the innate and adaptive immunity systems in spondyloarthropathies. IFN-γ, interferon γ; TNF-α, tumor necrosis factor α; TLR, Toll-like receptor; MSC, mesenchymal stem cell; IL, interleukin; PGE2, prostaglandin E2; M-CSF, macrophage colony-stimulating factor; TGFβ1, transforming growth factor β1;HLA-G5, human leukocyte antigen G5; DC, dendritic cell; NKT, natural killers; Treg, regulatory T cell, IDO, indolamine.

Spondyloarthropathies (SpA) are a group of inflammatory rheumatoid diseases which traditionally include ankylosing spondylitis (AS), psoriatic arthritis (PsA), reactive arthritis (ReA), arthritis associated with Crohn’s disease and ulcerative colitis as well as undifferentiated spondyloarthropathies. Apart from typical symptoms within the locomotor system, such as chronic inflammation of spinal joints, inflammation of entheses and inflammation of peripheral joints, the very complex clinical picture of SpA includes numerous non-articular manifestations, including the skin, intestines and eyes [1]. Local inflammatory changes in the skeletal system in the course of SpA result in local loss of bone tissue and the formation of erosions with simultaneous bone formation, which leads to profound destruction and impairment of the affected joints. Considerable progress has been made in recent years in the treatment of SpA thanks to the introduction of the tumor necrosis factor-α (TNF-α) inhibitors as well as interleukin 17 (IL-17) and interleukin 12/23 (IL-12/23) inhibitors [2,3,4]. Although non-articular symptoms can be well-controlled thanks to modern biological therapies, which considerably slow down the progression of destructive processes in the locomotory system, they do not affect changes in the osteo-articular system already present, nor do they inhibit the SpA-related bone-formation processes. Therefore, mesenchymal stromal cells, mesenchymal stromal cells (MSC), with their immunomodulatory and regenerative potential [5] (Figure 1), may represent a promising tool in long-term treatment of SpA, changing the present therapeutic approach.

## 2. The Role of Mesenchymal Stromal Cells in the Inflammatory Process and in the Pathogenesis of Spondyloarthropathies

### 2.1. Origin of Stromal Cells

MSC are able to form clones, to differentiate in multiple directions and to self-regenerate [6]. In early cultures, MSC resemble fibroblast (MSC type I) in their appearance and in the way they grow; round, small, self-regenerating cells are observed less frequently [7]; in later phases, MSC can be bigger and flatter (MSC type II) [8]. Unexpectedly, MSC are not immortal—they age and die after several passages [9]. Since MSC are present in many embryonic tissues (embryonic stem cells, ESC) and in adult individuals (adult stem cells, ASC), there are many methods of acquiring them. Embryonic stem cells can be collected after delivery from the umbilical cord blood, from Wharton’s jelly, from the placenta, amniotic fluid and as well as from subamniotic membrane and perivascular area of the umbilical cord. MSC has been identified in the following tissues in adult individuals: In marrow, in adipose tissue, in the skin, lungs, dental pulp, periosteum, skeletal muscles, tendons and synovial membrane [10], but clinical application of “adult” MSC is limited mainly to bone marrow-derived mesenchymal stromal cells (BM-MSC) and adipose-derived stem cells (ADSC, ASC) [11]. The International Society for Cellular Therapy (ISCT) has developed the minimum criteria to be used in identifying mesenchymal cells. By these assumptions, characteristic features of mesenchymal cells include the ability to adhere to a plastic base, the presence of three surface antigens: CD105 (endoglin), CD90 (Thy-1), CD73 (ecto-5′-nucleotidase) and concomitant absence of antigens CD45, CD34, CD14 or CD11a, CD79a, or CD19 and class II HLA, and the capability of in vitro differentiation towards three cellular lines: osteoblasts, chondroblasts and adipocytes [12]. A detailed description of stem cells includes additional information, such as the cell origin (tissue, organ, systemic), culture conditions, medium composition, presence of other antigens of positive identification and absence of negative markers, potential for differentiation, cloning, proteomes, secretomes and transcriptone data [13]. In vivo, MSC probably constitute a significant element of a niche of hematopoietic stem cells (HSC) [14], they take part in angiogenesis and regulation of blood vessel function [15] as well in controlling inflammatory processes [16].

### 2.2. The Role of Toll-Like Receptors in Activity of Stem Cells

Signal transfer in the inflammatory response of the innate immune system is effected, inter alia, by means of Toll-like receptors (TLR), which activate phagocytes. In cell culture studies, expression of various Toll-like receptors has been observed, including TLR3 (virus dsRNA receptor) and TLR4 (lipopolysaccharide receptor, LPS) [17]. In in vitro studies, under hypoxic conditions, short-term stimulation of human MSC by pro-inflammatory cytokines, such as interferon-γ (INF-γ), TNF-α, INF-α, IL-1β, increased expression of TLR1, TLR2, TLR3, TLR4, TLR5 [18], whereas prolonged stimulation resulted in a decreasing the number of TLR2 and TLR4 [19] and decreasing the inflammatory response. An increase in the expression of TLR3 and TLR4 on MSC observed in a study by Raicevic et al. boosted the response to LPS and poly(I:C) (polyinosinic-polycytidylic acid), which resulted in a decrease in the immunosuppressive properties of MSC [18]. It has also been suggested that MSC can acquire a pro-inflammatory phenotype (MSC1) when stimulated by TLR4 and undergo anti-inflammatory polarization (MSC2) when activated by TLR3 [20], which could partly explain the apparently conflicting roles of MSC in the inflammatory process. There is data which indicates the importance of TLR dysregulation in intensifying the inflammatory condition in spondyloarthropathy. Heuschen et al. examined patients with ulcerative colitis and described an increase in expression of TLR5 in patients with intensified inflammation of the intestinal mucosal membrane and a decrease in the number of TLR3 receptors in a healthy mucosal membrane with local suppression of the inflammatory condition [21]. An increase in TLR4 expression on peripheral blood mononuclear cells (PBMCs) in AS patients has been reported by de Rycke et al. [22] and by Yang et al. [23]. An increase in expression of TLR2 and TLR4 has also been observed in the synovial membrane collected from patients with other SpAs, including with PsA and undifferentiated SpA, compared to patients with rheumatoid arthritis (RA) and osteoarthritis (OA) [22]. Treatment with TNF-α inhibitors decreased the number of TLR2 and TLR4 receptors, both on peripheral mononuclear cells and on synoviocytes [22]. A small study by Candia et al. on PsA patients showed a temporary increase in the number of TLR2 on immature dendritic cells in vitro [24], whereas Myles et al. examined patients with juvenile chronic arthritis associated with enthesitis, and observed an increased expression of TLR2 and TLR4 on monocytes in peripheral blood and in articular fluid, which was associated with increased production of IL-6 and metalloproteinase 3 (MMP-3) following stimulation with LPS [25]. These studies indicate that there is a link between high expression of TLR in SpA, but they do not confirm a causal relationship between them. Expression of TLR in SpA may intensify the inflammatory response or be a specific indicator of chronic inflammation.

### 2.3. Stem Cells at an Early Phase of Inflammation

The immunomodulatory activity of MSC in an early phase of the inflammatory process seems to favor the development of an effective immune response. In a study on mice, a MSC response associated with recognition of bacterial proteins resulted in an increased secretion of IL-6, IL-8, GM-CSF (granulocyte-macrophage colony-stimulating factor) and MIF (macrophage migration inhibitory factor)—which are factors stimulating influx and activity of neutrocytes [26]. In a study conducted by Mantovani et al., BM-MSC activated through the TLR3 receptor extended the survival period of neutrophils—inactive and activated by IL-6, INF-γ and GM-CSF [27]. In addition, MSC can produce chemokines (CXCL-9, CXCL-10 and CXCL-11) by stimulating recruitment of lymphocytes to the inflammation sites [28]. Such an effect has been observed in in vitro studies in mouse and human MSC cultures at low concentrations of TNF-α and INF-γ, where human MSC reduced secretion of IDO in these conditions, and mouse MSC produced decreased amounts of iNOS, which was associated with decreased inhibition of T cell proliferation [29,30]. The findings of these studies may suggest an effect of concentrations of IDO and iNOS on the pro- and anti-inflammatory activity of human and murine MSC, respectively. Through expression of ligands (C-C motif) of chemokines CCL2, CCL3, CCL12, human and murine BM-MSC can boost influx of monocytes to the inflammation sites, thereby supporting local regenerative processes [31].

### 2.4. Monocytes and Macrophages

**Figure 2:**
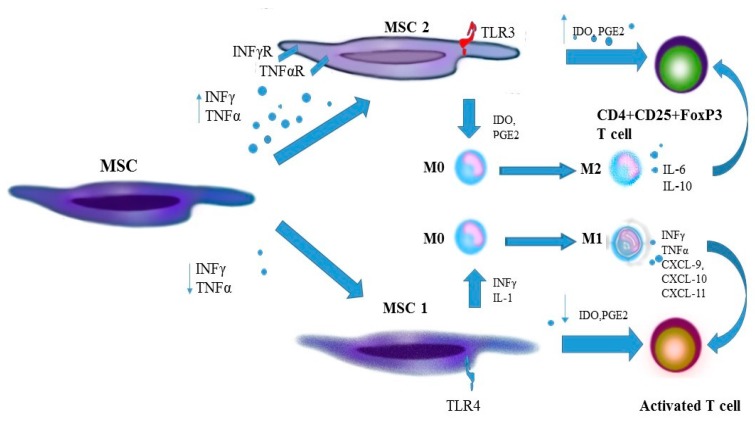
Polarization of MSC into an anti-inflammatory and pro-inflammatory phenotype and impact of anti-inflammatory and pro-inflammatory MSC on T cells activity. IFN-γ, interferon γ; TNF-α, tumor necrosis factor α; TLR, Toll-like receptor; MSC, mesenchymal stem cell; IL, interleukin; PGE2, prostaglandin E2; IDO, indolamine M, monocyte; CXCL, chemokine.

Apart from recruiting circulating monocytes, MSC can affect the function of macrophages at inflammation sites. It seems that polarization of macrophages towards a pro-inflammatory M1 phenotype and an anti-inflammatory M2 phenotype can depend on the immunomodulatory properties of MSC [32,33]. MSC polarize M0 macrophages to the M1 phenotype at low concentrations of IL-6. Increased production and secretion of pro-inflammatory cytokines by M1 macrophages and activated T cells stimulate MSC to produce mediators, including immunosuppressive agents, such as iNOS (inducible NO synthase) in cultures of murine MSC and IDO (indolamines) [34] (Figure 2). In studies of joint cultures of monocytes and human or murine BM-MSC, polarization of macrophages to the anti-inflammatory M2 phenotype depended on the cellular interactions and on E2 prostaglandin (PGE2) concentrations and on products of IDO activity, including kynurenine (a product of tryptophan degradation) and other catabolites [35]. Activation of MSC by TNF-α and IFN-γ as well as LPS boosts expression of cyclooxygenase 2 (COX2) and IDO in BM-MSC, additionally stimulating macrophage activation to the M2 phenotype [36]. M2 macrophages produce mainly anti-inflammatory cytokines IL-10 and TGF-β and small amounts of pro-inflammatory cytokines IL-1, IL-6, TNF-α and IFN-γ, thereby inhibiting the inflammatory process and helping to regenerate damaged tissues [27]. Polarization of monocytes and macrophages to the pro- or anti-inflammatory phenotype in SpA may be responsible for an active inflammatory process, regeneration processes and rebuilding the affected tissues. Zhao et al. examined peripheral blood in patients with advanced AS and detected significant polarization of monocytes to the M2 type, with the M2/M1 ratio being correlated positively with the damage to the affected structures, and negatively with inflammation indicators (ESR, CRP) and BASDAI (Bath Ankylosing Spondylitis Disease Activity Index) [37]. Other researchers have also described polarization of histiocytes to the M2 type at sites affected by inflammation in AS [38] and PsA [39]. Interestingly, a therapy with TNF-α inhibitors in SpA is linked with an increase in the M2/M1 ratio, which could be attributed to a decrease in the number of M1 monocytes [37], but it does not prevent progressive bone formation, typical of SpA [40]. Guihard et al. found stimulation of MSC differentiation towards osteoblasts by activated monocytes is effected in the presence of OSM (oncostatin M), an IL-6 cytokine, and is mediated through a type II receptor on MSC, which activates the transcriptive agent STA3 [41].

### 2.5. Dendritic Cells

Studies of animal models and human dendritic cells (DC) in SpA provide data which indicates a contribution of DC in the development of SpA. DC HLA-B27^+^ are capable of synthesis of IL-23, which is one of the main pro-inflammatory cytokines in SpA [42,43]. IL-23 exerts a systemic effect through induction of differentiation of naive T cells in lymph nodes to pro-inflammatory Th17 [44] and through stimulation of lymphocytes IL-23R^+^ residing in entheses to secrete IL-22 and to stimulate osteoblasts, leading to local bone formation [45]. MSC inhibit differentiation of CD14^+^CD1a precursors originating in peripheral and umbilical blood to dendritic cells [46]. Zhang et al. found the presence of MSC to be associated with reduced expression of presenting and co-stimulating cells, including CD1a, CD40, CD80, CD86 and HLA-DR during the process of DC differentiation and limited expression of CD40, CD86 and CD83 during DC maturation [47]. Similar findings have been presented by Jiang et al., where the presence of MSC additionally decreased expression of CD83 on already-matured DC, which suggested the loss of maturity features by dendritic cells [48]. Through secreted PGE2, MSC can also inhibit maturation of DC stimulated by CSF and IL-4 without disrupting the process of DC maturation stimulated by LPS [49]. An effect has been described of MSC resulting in a decrease in DC activity in antigen transformation and presentation to T cells, related to inhibiting of MAPKs (mitogen-activated protein kinases) activity following stimulation of by TLR4 [50]. In a recently published study, MSC in a cell culture polarized DC to a regulatory phenotype with expression of IL-6 and IL-10 [51].

### 2.6. Neutrophils

Neutrophils are a valuable source of IL-17, which is another pro-inflammatory cytokine of key importance in the pathogenesis of SpA. Appel et al. examined facet joints in patients with axial SpA and noted that it was mainly neutrophils that were responsible for local synthesis of IL-17 [52]. It seems that neutrophils are stimulated by MSC, which may maintain the inflammation. Maqbool et al. presented the findings of a study in which MSC extended the survival period of neutrophils deprived of nutrients or plasma [53]. In a study conducted by Raffaghello et al., MSC secreted IL-6, whereby they were able to inhibit apoptosis of resting neutrophils and those activated with IL-8 [54]. In another study, MSC activated by TLR3 significantly boosted the vitality and activity of neutrophils through IL-6, IFN-γ and GM-CSF [28].

### 2.7. NK Cells

Natural killer cells are one of the main parts of the innate immune system. The discovery that the HLA-B27 antigen is specifically recognized by the inhibitory KIR3DL1 receptor of NK cells and identifying the link between the expression of KIR activating and inhibitory receptors with the activity of AS indicates that NK may play a significant role in pathogenesis of SpA [55]. MSC can change the NK phenotype and inhibit their proliferation, as well as the secretion of cytokines and cytotoxicity against T cells with expression of class I HLA. This activity is exerted through intercellular interactions or soluble mediators, such as TGF-β1 and PGE2 [56]. MSC can inhibit IL-2-stimulated proliferation of inactive NK [57]. Through HLA-G5, MSC have an inhibitory effect on NK-dependent cytolysis and on INF-γ secretion [58]. In a study by Prigione et al., MSC inhibited INF-γ production through activated NK with no effect on their cytotoxic activity [59].

### 2.8. T Cells

MSC have a modulatory effect on proliferation of T cells by the production and secretion of TGF-β, hepatocyte growth factor (HGF), PGE2, IDO and HO (hemoxygenase) [60]. Human MSC inhibit the proliferation of T cells, both CD4^+^ and CD8^+^ also with IDO, while at the same time inducing proliferation of regulatory T cells (Treg) [61]. The inhibitory effect of MSC on T cells decreases when there are no monocytes present, which indicates not only an effect of soluble factors secreted by MSC, but it also suggests cellular interdependence of MSC and monocytes in inhibiting lymphocyte proliferation [62]. It appears that MSC inhibit differentiation of effector Th17 [63], although the mechanisms affecting it are not clear [64,65]. Huang et al. described an inhibitory effect of human umbilical cord derived MSC (hUCMSC) on T cells in SpA patients. In a culture with mononuclear cells from peripheral blood, hUCMSC considerably reduced IL-17 production, which may suggest a therapeutic potential of MSC [66]. Th17 cells play a key role in development of an inflammatory condition which accompany SpA, they recruit circulating monocytes and neutrophils to the sites affected by the disease, stimulate maturation of osteoclasts, and, in consequence, resorption of bone tissue [67,68]. The ability of MSC to convert mature Th17 into Treg is very important in the context of chronic inflammation in SpA [69,70]. Treg cells are mediators of immune tolerance which exert their effect through suppression of effector T cells and inhibition of tissue destruction induced by an immune process. Examination of peripheral blood and articular fluid of patients reveals a relative reduction in the number of Treg cells [71,72] and recent studies have shown a link between functional defects of CD4^+^CD25^high^FoxP3^+^ [73] and the Treg/Th17 balance being disturbed with the development of SpA [74]. An ability to induce proliferation of Treg, which has been confirmed in numerous studies, is one of the key mechanisms of limiting inflammation by MSC. Joint culturing of MSC and peripheral blood mononuclear cells (PBMC) stimulated differentiation of CD4^+^ cells towards Treg cells with the expression of CD25^high^FoxP3^+^ [75]. In cultures of MSC and washed CD4^+^ cells or PBMC with monocyte depletion did not show any differentiation of lymphocytes towards Treg cells, whereas proliferation of CD4^+^CD25^high^FoxP3^+^ cells in cultures took place after monocytes were added [76]. Induction of Treg cells dependent on MSC may be linked to the secretion by MSC of the soluble human leukocyte antigen G5 (sHLA-G5). The HLA-G5 molecule inhibits the proliferation of alloreactive T cells and stimulates differentiation of immature T cells towards suppressor Treg cells [77] and is linked to the induction of proliferation of CD4^+^CD25^high^Fox P3^+^ cells [78]. In a study conducted by Wu et al., BM MSC in AS patients had decreased immunomodulatory potential; in addition, an increased amount of Treg and Fox P3^+^ cells was found, as well as an increased amount of T cells with CCR4^+^CCR6^+^ receptors compared to healthy people. This may suggest a decreased immunomodulatory potential of MSC as a factor which plays a role in the development of AS [74].

### 2.9. B Cells

There is currently no proof of the participation of specific antibodies in the pathogenesis of spondyloarthropathy, but one must bear in mind that B cells have chemotactic properties, they produce cytokines and can be very effective antigen-presenting cells [79]. With their immunomodulatory potential, regulatory B cells (Breg) can also inhibit Th1 response and differentiation of Th17 cells [80]. An increased number of circulating Breg cells in SpA has been reported [81] and, although no link has been found with disease activity, the number of Breg cells has been reported to decrease in patients treated with anti-TNF-α [82]. MCS regulate a number of functions of B-cells. In a study conducted by Corcione et al., MSC inhibited proliferation of B-cells by arresting the cellular cycle at the G0/G1 phase and secretion of immunoglobulins (Ig) IgM, IgG and IgA, which was reflected by inhibited differentiation of lymphocytes. In the same study, expression of chemokine receptors (C-X-C motif) CXCR4 and CXCR5 as well as CCR7 on B-cells decreased considerably in the presence of MSC, which may suggest an effect of MSC on the chemotactic properties of B cells [83]. Lee et al. described inhibition of IgG production by a C3 component of the complement secreted by MSC following infection by a strain of *Mycoplasma arginini* [84]. In a different study, MSC, following stimulation by TLR4, exhibited increased expression of the B-cell activating factor (BAFF), thereby affecting immunoglobin production [85]. In another study, excitation of MSC by INF-γ stimulated cells to secrete galectin 9 (Gal-9), an inhibitor of T- and B-cell proliferation and production and secretion of antigen-specific antibodies [86]. However, different findings were reported by Rosado et al. and by Ji et al., who described increased proliferation and differentiation of B cells in the presence of BM-MSC and umbilical cord MSC (UC-MSC), respectively [87,88]. These discrepancies can probably be attributed to an indirect effect of other factors present in the cultures, which were not covered by those studies.

## 3. The Role of Stem Cells of Irregular Ossification in Spondyloarthropathy

It appears that MSC in SpA are involved in processes of irregular ossification. MSC can affect the process of bone mineralization by regulating the activity of TNAP (tissue-nonspecific alkaline phosphatase). In a study which sought to provide a probable explanation of the differences between changes in bones observed in RA and SpA, Ding et al., treated cultured human MSC (hMSC) with TNF-α and IL-1β. The action of these cytokines resulted in decreased expression of collagen and increased activity of TNAP. Differences in the effect of TNF-α and IL-1β on expression of collagen and the activity of TNAP can partially explain why bone changes in SpA are linked to bone loss and accompanying bone formation, whereas they are linked to the formation of corrosions in RA [89]. In another study, stimulation of osteoblast activity with Wnt5a was observed in response to the action of TNF-α. The concentration of Wnt5a was significantly increased by TNF-α and it was linked to an increase in the activity of TNAP and intensified mineralization. The findings of this study indicate a connection between inflammation in SpA and bone formation by activation of the cannonical Wnt/β-catenin pathway by Wnt5a. Stimulation of ossification by MSC could explain the lack of, or weak, effect of an anti-TNF-α therapy in inhibiting bone formation in SpA [90]. Characteristic features of all SpAs include inflammatory changes in entheses, which are independent of inflammation of synovial membrane in joints. MSC in places where ligaments, tendons and articular capsules are attached to bones can be a reservoir of cells responsible for the repair of articular cartilage—which is a tissue of a low regenerative potential—damaged by inflammation [91]. In a study on a rat model of the degenerative joint disease, regeneration of articular cartilage was faster and of a better quality following intra-articular injections of MSC compared to the administration of mature chondrocytes [92]. Differentiation of MSC in entheses towards tenocytes, chondrocytes or osteoblasts depends, inter alia, on the tensile force [93]. Under the influence of mechanical stimulae, mechanosensitive calcium permeable channels become involved in changes in intracellular calcium concentrations [94,95]. Stimulation of these channels in the MSC membrane, which results in MSC activation, can trigger inflammatory processes and ossification in entheses, which confirms the hypothesis of the role of physical damage in the development of SpA [96,97]. Apart from the mechanical load of the structures of entheses, osteogenic differentiation of MSC is stimulated by fibronectin, whereas a high concentration of type I collagen inhibits osteoblastogenesis and promotes differentiation towards tenocytes [93]. In a recently published study by Xie et al., differentiation of MSC towards osteoblasts in AS patients was linked to disturbed balance between bone morphogenic protein-2 (BMP-2) and Noggin protein. The discovery of this mechanism, which leads to intensified osteogenesis in entheses, suggests that restoring the BMP-2/Noggin balance or local suppression of MSC could inhibit excessive bone formation in SpA [98]. 

Numerous publications have confirmed the immunomodulatory effect of MSC on elements of the inflammatory process. There is plenty of data which may indicate the role of MSC in spondyloarthropathies (Table 1), which encourages further studies on applications of MSC in the treatment of SpA.

## 4. The Role of MSC in the Treatment of Spondyloarthropathies

The available data on the immunomodulatory effect of MSC comes mainly from in vitro studies. However, there has been a lot of data from in vivo studies which confirms such an effect of MSC. Adipose-tissue-derived MSCs (AT-MSC) effectively suppressed the T1-dependent immune response and stimulated the proliferation of Treg in transgenic diabetic NOD/SCID mice, in effect maintaining the function of β cells in the pancreas [99]. Monocytes incubated in the presence of AT-MSC administered by infusion decreased the activity of chronic intestine inflammation and protected against the development of severe sepsis by inducing immunomodulatory macrophages secreting IL-10 and inhibiting uncontrolled production of inflammatory mediators [100]. Improvement of survival and mitigation of the course of sepsis following IV administration of MSC and their interaction with monocytes and macrophages was also described in the paper by Nemeth et al., which was linked to the production of IL-10 by monocytes and macrophages and decreased serum concentrations of pro-inflammatory TNF-α and IL-6 [36]. In other studies, MSC improved the survival of skin grafts [101], allogenic corneal transplants [102] and alleviated symptoms of experimental encephalomyelitis in mice [103]. Administration of human BM-MSC, UC-MSC and AT-MSC in asthma increased the pool of macrophages in pulmonary alveoli, mitigated bronchial hyper-reactivity, reduced eosinophil counts in bronchi and the production of Th2-dependent cytokines. Depletion of macrophages in pulmonary alveoli resulted in intensification of bronchial hyper-reactivity [104]. The immunomodulatory effect of MSC seems not to result from direct intercellular interactions or cells colonizing specific organs, but from secreted soluble mediators, which affects the systemic effect of MSC. This was confirmed in a study conducted by Zanotti et al., in which polymer encapsulated MSC (E-MSC) exerted an immunosuppressive and anti-inflammatory effect, probably by means of secreted soluble agents [105]. 

The potentially regenerative and immunomodulatory properties of MSC in arthritis and in degenerative joint disease have also been studied [106,107]. The first reports of the effectiveness of treatment of autoimmune diseases come from a description of bone marrow transplants in patients with comorbidities, such as proliferative diseases of the hematopoietic system and autoimmune diseases [108]. A positive outcome of bone marrow transplant on the course of immune diseases encouraged researchers to make numerous attempts to apply HSC and MSC in RA, systemic lupus erythematosus (SLE), scleroderma and sclerosis multiplex [108]. Unfortunately, no studies have been conducted of the efficacy of SpA treatment with stem cells. There have been several reports in the literature on bone marrow transplants for hematological reasons in patients with psoriatic arthritis and ankylosing spondylitis. Remission and even a reduction of radiographic changes has been achieved in the patients [109,110,111,112,113]. In 2012, the first autologous HSC transplant was carried out following chemotherapy in a male patient with AS and with the HLA-B27 antigen, with the intent to treat ankylosing spondylitis. A complete remission was achieved, which lasted throughout the two-year follow-up period [114]. In another study, Wang et al. described the effectiveness of IV administration of allogenic MSC in 31 AS patients, following ineffective treatment with NSAIDs. The study lasted 20 weeks, MSC infusions were carried out four times, on days 0, 7, 17 and 21. At the end of the fourth week, a response to treatment was achieved, as assessed by ASAS 20 (Assessment in Ankylosing Spondylitis Response Criteria 20), in approx. 75% of the patients, a reduction of ASDAS-CRP (Ankylosing Spondylitis Disease Activity Score Containing C-Reactive Protein) from 3.6 ± 0.6 to 2.4 ± 0.5 was recorded with an increase to 3.2 ± 0.8 in the 20th week. The response to treatment lasted 7.1 weeks on average. No adverse effects were reported in the study [115]. There are several clinical trials currently underway to assess the efficacy and safety of stem cell transfusions in AS [116,117,118,119] (Table 2). 

## 5. Conclusions

Promising results of studies into the application of stem cells in autoimmune diseases may be indicative of the therapeutic potential of MSC in SpAs. Depending on conditions in joints, MSC can exhibit anti-inflammatory or pro-inflammatory activity and can speed up regeneration in entheses or contribute to their ossification, which is typical of SpA. Local modification of MSC activity in the anti-inflammatory direction by appropriate agents or the administration of selected MSC may prove a highly affective option in the treatment of severe forms, especially in ankylosing spondylitis and psoriatic arthritis. However, it is still uncertain whether MSC used in SpA therapy should be autologous or allogenic and which tissue origin of cells is the most beneficial. It is also unclear whether treatment should be applied in early stages of a disease or rather as a regenerative therapy and which route of administration should be chosen, the number of cells and the therapeutic regimen. Obviously, further studies will be needed before the use of MSC in SpA could become the treatment of choice. 

## Figures and Tables

**Table 1:** An analysis of a potential role of stem cells in the development of spondyloarthropathy.

Elements of Pathogenesis of Spondyloarthropathy	Results of Stem Cell Action
Dysregulation of TLR. Increase in expression of TLR2 and TLR 4 on mononuclear cells of peripheral blood and in articular synovial membrane [21,22,23,24].	Acquisition of the pro-inflammatory phenotype by MSC following stimulation by TLR4 and the anti-inflammatory phenotype following stimulation by TLR3 [18,19,20].
Increased production of pro-inflammatory TNF-α and IFN-γ by activated monocytes and macrophages.	Activation of MSC with TNF-α and IFN-γ boosts expression of iNOS, COX2 and IDO and favours polarisation of monocytes and macrophages to the anti-inflammatory M2 phenotype M2 [34,35,36].
Increase in production of inflammatory cytokines, e.g., IL-12, IL-23, IL-6 by dendritic cells [42,43].	Inhibition of differentiation of precursors of CD40CD1a into DC, inhibition of the ability to present antigen by DC, induction of the loss of maturity features by DC [46,48,49].
Increase in local production of IL-17 in joints by neutrophils [52].	Inhibition of apoptosis and stimulation of activity of activity of neutrophils by IL-6, IL-8 IFN-β and GM-CSF [28,54].
A link between expression of activating KIR receptors on NK cells with the disease activity. Recognising of HLA B27 antigen by the KIR3DL1 receptor [55].	Inhibition of proliferation, cytokine secretion and cytotoxicity of NK cells [56,57,58,59].
The key role of Th17 cells in development of SpA [67,68]	Ability of mature Th17 to convert into Treg [69,70].
Decrease in the amount of Treg. Upsetting the Treg/Th17 balance. Functional defects of CD4^+^CD25^+^FOXP3 [71,72,73,74].	Induction of Treg proliferation. Stimulation of differentiation of CD4 towards CD4^+^CD25^+^FOXP3 [75].
Ossification of entheses, formation of new bone tissue on marginal surfaces of joints [1].	Regulation of ossification with TNAP. Increased bone formation by activation of Wnt/β-catenin pathway with Wnt5a. Ossification of entheses following stimulation of calcium channels in MSC by mechanical stimuli [89,90,97].

TLR, Toll-like receptor; TNF-α, tumor necrosis factor-α; IFN-γ, interferon γ; iNOS, inducible NO synthase; COX2, cyclooxygenase 2; IDO, indolamine; IL, interleukin; GM-CSF, granulocyte-macrophage colony-stimulating factor; DC, dendritic cells; NK, natural killers; TNAP, tissue-nonspecific alkaline phosphatase.

**Table 2:** Use of stem cells in patients with spondyloarthropathies in published literature and registered clinical trials.

SpA	Stem Cells	Description	Reference
Psoriatic arthritis	Allogenic blood stem cell transplantation (myeloablative)	Concomitant chronic myelogenous leukemia. Graft versus autoimmunity effect.	Slavin et al. [109]
Psoriatic arthritis	Allogenic hematopoetic stem cell transplantation	Concomitant aplastic anemia. Short remission with long chronic disability-free period	Woods et al. [110]
Psoriatic arthritis	Autologous hematopoetic stem cell transplantation (myeloablative)	Concomitant multiple myeloma. Complete remission of arthritis and skin lesions	Braiteh et al. [111]
Ankylosing spondylitis	Autologous hematopoetic stem cell transplantation	Concomitant lymphoma. The patient underwent chemotherapy. Clinical remission for both AS and lymphoma	Jantumen et al. [112]
Ankylosing spondylitis	Allogenic blood stem cell transplantation	Concomitant acute myeloid leukemia. The patient underwent chemotherapy and body irradiation. Clinical remission. Partial radiological regression of syndesophytes	Britanova et al. [114]
Ankylosing spondylitis	Autologus hematopoetic stem cell transplant	The first reported intentional stem cell transplant for AS. The patient underwent chemotherapy. Complete remission for AS for two-year follow up period	Yang et al. [113]
Ankylosing spondylitis	Allogenic mesenchymal stem cells intravenous infusion	Trial involving 31 AS patients. No adverse effects noted. Reduction of ASDAS-CRP from 3.6 ± 0.6 to 2.4 ± 0.5 at the 4th week. The percentage of ASAS 20 responders reached 77.4%	Wanga et al. [115]
Ankylosing spondylitis	Human umbilical cord-derived mesenchymal stem cells	Clinical trial. Phase 1. Human umbilical cord-derived MSCs at a dose of 1.0 × 10^6^ MSC/kg, repeated after three months and DMARDs such as sulfasalazine, methotrexate, thalidomide for 12 months	Clinical Trials. gov Identifier: NCT01420432 [116]
Ankylosing spondylitis	Human mesenchymal stem cells	Clinical trial. human mesenchymal stem cells: 1.0 × 10^4-6^ cells/kg, IV on day 1 of each 14–60 day cycle, 1–6 times treatment, plus NSAIDs.	ClinicalTrials.gov Identifier: NCT01709656 [117]
Ankylosing spondylitis	Human bone marrow-derived MSCs	Recruiting clinical trial. Phase 2. hBM-MSCs at a dose of 1.0 × 10^6^ MSC/kg, receive infusion per week in the first 4 weeks and every two weeks in the second 8 weeks. Study Start Date: June 2016 Estimated Study Completion Date: December 2018	ClinicalTrials.gov Identifier: NCT02809781 [118]
Ankylosing spondylitis	Mesenchymal stem cells	Clinical trial. Phase I/II. To observe the safety and clinical effect of MSC transplantation in AS	Clinical trial. Registration number: ChiCTR-TRC-11001417 [119]

AS, ankylosing spondylitis; ASDAS-CRP, Ankylosing Spondylitis Disease Activity Score Containing C-Reactive Protein; ASAS 20, Assessment in Ankylosing Spondylitis Response Criteria 20; hBM-MSCs, human bone marrow-derived mesenchymal stem cells, DMARDs, disease-modifying anti-rheumatic drugs; NSAIDs, on steroidal anti-inflammatory drugs.
